# Efficacy, Safety and Patient-Reported Outcomes with Preservative-Free (PF) Tafluprost or PF-Dorzolamide/Timolol Compared with Preserved Latanoprost: A Prospective Multicenter Study in Korean Glaucoma Patients with Ocular Surface Disease

**DOI:** 10.3390/ph15020201

**Published:** 2022-02-07

**Authors:** Sang-Woo Park, Jiwoong Lee, Michael S. Kook

**Affiliations:** 1 Chonnam National University Hospital, Gwangju 61469, Korea; exo70@jnu.ac.kr; 2 Pusan National University Hospital, Busan 49241, Korea; glaucoma@pusan.ac.kr; 3 Department of Ophthalmology, Asan Medical Center, Seoul 05505, Korea

**Keywords:** dorzolamide/timolol, glaucoma, P-latanoprost, ocular surface disease, preservative-free, tafluprost

## Abstract

To compare the efficacy, patient-reported satisfaction, and safety of preservative-free (PF)-tafluprost, PF-dorzolamide/timolol and preservative-containing (P)-latanoprost in Korean glaucoma patients with ocular surface disease (OSD). In a multicenter, prospective, interventional, non-randomized, controlled 12-week trial, 107 eligible patients received PF-tafluprost (*n* = 37), PF-dorzolamide/timolol (*n* = 34), or P-latanoprost eye drops (*n* = 36). Outcomes included changes from baseline in OSD Index (OSDI) scores (primary endpoint), intraocular pressure (IOP), and patient-reported treatment satisfaction, and safety at 12 weeks. At 12 weeks, the mean total OSDI and subdomain (dry eye symptoms, visual-related function, environmental triggers) scores significantly improved from baseline with PF-tafluprost and PF-dorzolamide/timolol, but not with P-latanoprost. Significantly more PF-tafluprost than P-latanoprost recipients reported ‘highly improved/improved’ satisfaction (no significant difference between PF-dorzolamide/timolol and P-latanoprost). IOP changes were comparable among all three treatment groups. No new safety concerns were observed. PF-tafluprost and PF-dorzolamide/timolol showed statistically and clinically significant reductions in OSDI compared with P-latanoprost in Korean glaucoma patients with OSD.

## 1. Introduction

Glaucoma is a heterogeneous group of progressive optic neuropathies, characterized by damage to the optic nerve, which cause visual field defects [1]. Globally, glaucoma is a leading cause of irreversible blindness [2]. Risk factors for the most common form of the disease, primary open-angle glaucoma, include older age, increased intraocular pressure (IOP), sub-Saharan African ethnic origin, family history, and high myopia. Risk factors for primary angle-closure glaucoma include older age, hyperopia, and East Asian ethnic origin [1].

Currently, the only effective proven treatment for glaucoma is a reduction in IOP. Topical medications used to lower IOP include prostaglandin analogs (PGAs; latanoprost, tafluprost, travoprost, unoprostone, bimatoprost), β-adrenergic blockers (timolol, levobunolol, carteolol, metipranolol, betaxolol), and carbonic anhydrase inhibitors (dorzolamide, brinzolamide) [1,3]. Because glaucoma has a chronic nature and vision loss is irreversible, treatment for glaucoma is lifelong. However, daily long-term IOP treatment can cause ocular surface toxicity. Ocular surface disease (OSD) is a multifactorial disease, which is characterized by tear film instability, inflammation, and tear hyperosmolarity [4,5]. The prevalence of OSD in glaucoma patients is high, with estimates ranging from 39% to 59% [6,7,8]. Higher (i.e., more severe) ocular surface disease index (OSDI) scores have been reported in patients with glaucoma receiving topical IOP-lowering medical treatments, particularly those using preservative-containing anti-glaucoma eye drops with a longer treatment duration, as well as those using multiple IOP-lowering medications [6,9,10,11,12].

Ocular surface toxicity associated with the long-term use of topical anti-glaucoma medication can result from the active ingredient(s), especially for patients with pre-existing OSD, and/or from preservatives and excipients [13,14,15,16,17,18]. Most eye drops contain preservatives, commonly benzalkonium chloride (BAK), which can cause corneal, conjunctival, and trabecular meshwork toxicities resulting in OSD [15,16,17,18,19,20,21]. A range of deleterious effects in these tissues has been reported for BAK including apoptosis, oxidative stress, disruption of tight junctions, cytoskeleton changes, and induction of inflammatory chemokines [19,22,23]. There has also been a report of severe impairment of corneal sensitivity owing to chronic use of preserved glaucoma medication containing BAK, which indicates the wide-ranging impact of BAK in changing the ocular surface structure [18].

Since glaucoma requires long-term treatment, patients’ quality of life (QoL) should be considered as one of the key factors for the successful treatment of glaucoma. OSD in glaucoma patients is associated with poorer health-related QoL, with progressive vision loss, which is an important factor for the decline in QoL over time and has an impact on daily living activities [10,24,25,26,27,28,29,30].

The aims of the current study were to evaluate the OSDI using a questionnaire regarding the QoL of patients with glaucoma who were medically treated, and to identify an association between the OSDI in glaucoma patients and the use of different topical medications with or without preservatives. In this study, the effects of two preservative-free (PF) glaucoma medications—tafluprost (PF-tafluprost) and dorzolamide/timolol (PF-dorzolamide/timolol)—on OSD and patient satisfaction (using patient-reported outcomes [PRO]) were compared with those of preservative-containing glaucoma medication (P-latanoprost) in Korean glaucoma patients.

## 2. Results

Due to difficulties with recruitment and a lower-than-expected drop-out rate, patient enrollment was discontinued prior to reaching 120 participants. Overall, 109 glaucoma patients with OSD were recruited and screened, which satisfied the minimum number (*n* = 102) of participants needed for the current study (see Section 4.6); of these, 2 patients were excluded due to missing data and 107 patients were enrolled to PF-dorzolamide/timolol (*n* = 34), PF-tafluprost (*n* = 37), or P-latanoprost (*n* = 36) (FAS1) treatment groups. The FAS2 population (missing values not replaced) comprised 105 patients (two patients in the PF-tafluprost group were not included in FAS2). There were no between-group differences in patient demographics at baseline. Mean age was 62.1, 63.2, and 63.0 years, respectively, and 50.0%, 40.5%, and 38.9% of patients were male. Other than P-latanoprost, no patients received other eye drops at baseline. Oral anti-diabetic agents were the most common medication in the PF-dorzolamide/timolol group (12.7%), anti-anginal and dyslipidemic drugs in the PF-tafluprost group (10.5%), and anti-asthmatic/COPD and oral anti-diabetic drugs in the P-latanoprost group (10.4%) (Table 1).

### 2.1. Patient-Reported Symptoms and Quality of Life

At baseline, no significant between-group differences were observed in the OSDI total and domain scores. In the FAS1 population (Table 2), OSDI total scores were improved significantly by PF-tafluprost (−9.5; *p* = 0.0042) and PF-dorzolamide/timolol (−10.5; *p* = 0.0038) from baseline to 12 weeks (primary efficacy outcome). In contrast, the change from baseline in OSDI total score with P-latanoprost was not significant (−1.5; *p* = 0.6256). In addition, after 12 weeks, significant changes from baseline in each OSDI subdomain were found for both PF-tafluprost and PF-dorzolamide/timolol, but not for P-latanoprost (Table 2). Similar results were observed in the FAS2 (data not shown) and PPS (Supplementary Materials Table S1) populations.

At follow-up (FAS1), no significant differences between PF-tafluprost and P-latanoprost groups were found for mean OSDI total or domain scores (Supplementary Materials Table S2). In contrast, significant differences were found for mean OSDI total scores between PF-dorzolamide/timolol and P-latanoprost groups (*p* = 0.0426). Significant differences between these treatment groups were also observed for mean domain scores for visual-related function (*p* = 0.0110) and environmental triggers (*p* = 0.0226) (Supplementary Materials Table S2). Similar results were found in the FAS2 (Supplementary Materials Table S3) and PPS (Supplementary Materials Table S4) populations.

When comparing PF-tafluprost and P-latanoprost, PF-tafluprost showed a statistically significant improvement over P-latanoprost in the OSDI environmental triggers domain (FAS1; Table 3). In the FAS2 population, PF-tafluprost showed significant improvements over P-latanoprost in total OSDI score, as well as in the visual-related function and environmental trigger subscale domains (data not shown). Compared with P-latanoprost, PF-dorzolamide/timolol showed statistically significant improvements in the environmental trigger domain (FAS1, Table 3). Similar results were observed in the PPS population (Supplementary Materials Table S5).

**Figure 1:**
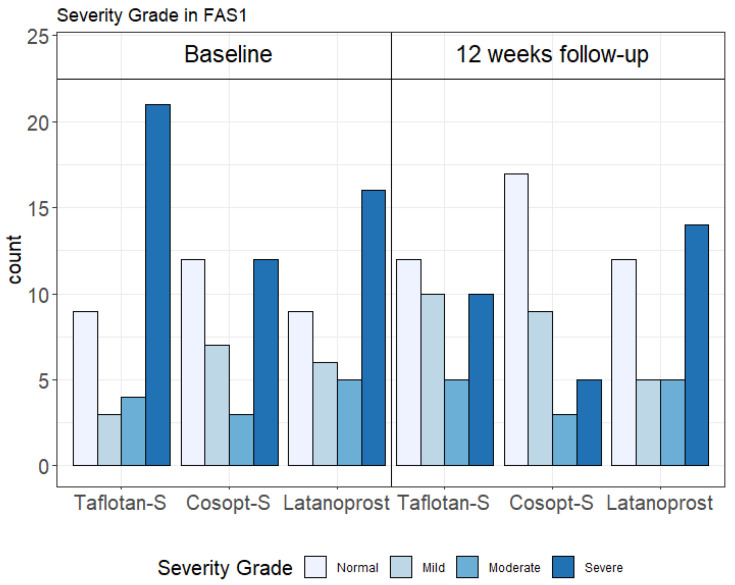
Ocular surface disease index (OSDI) severity categories in each treatment arm at baseline and 12-week follow-up in the Full Analysis Set 1 (FAS1) population (*n* = 107). Taflotan-S = preservative-free tafluprost; Cosopt-S = preservative-free dorzolamide + timolol; Latanoprost = preservative-containing latanoprost.

The proportions of normal OSDI scores (0–12) were increased by PF-tafluprost from 24.3% to 32.4%; by PF-dorzolamide/timolol from 35.3% to 50.0%; and by P-latanoprost from 25.0% to 33.3%. The proportions of severe OSDI scores (33–100) were reduced by PF-tafluprost from 56.8% to 27.0%; by PF-dorzolamide/timolol from 35.3% to 14.7%; and to a lesser extent by P-latanoprost, from 44.4% to 38.9% (FAS1 population, Figure 1). Similar trends were found in the FAS2 (data not shown) and PPS (Supplementary Materials Figure S1) populations.

In patients who self-assessed changes in treatment satisfaction (PRO) at 12 weeks (FAS2), PF-tafluprost produced a significantly higher rate of improvement in PROs (scored as improved/highly improved) compared with P-latanoprost (51.3% vs. 16.7%; *p* = 0.007). In contrast, the rate of PRO improvement with PF-dorzolamide/timolol was not significantly different to that achieved by patients receiving P-latanoprost (35.3% vs. 16.7%; *p* = 0.078) (Table 4). Similar results were found in the PPS population: a significantly higher rate of improvement in PROs was found for PF-tafluprost vs. P-latanoprost (*p* = 0.016), but not for PF-dorzolamide/timolol vs. P-latanoprost (*p* = 0.102) (Table 4).

### 2.2. Changes in IOP

Mean (SD) changes in IOP in the PF-tafluprost, PF-dorzolamide/timolol, and P-latanoprost FAS1 groups were −0.03 (2.76), 0.35 (3.66), and −0.03 (1.87) mmHg, respectively. No significant differences in change in IOP were found for PF-tafluprost vs. P-latanoprost (*p* = 0.999) and PF-dorzolamide/timolol vs. P-latanoprost (*p* = 0.590) (Table 5). Similar non-significant results were found in the FAS2 and PPS populations (data not shown).

### 2.3. Safety

For PF-tafluprost (*n* = 37), two AEs in two subjects (5.4%) were reported: eye pruritis (*n* = 1) and blurred vision (*n* = 1). Both cases were mild in intensity, possibly related to the study medication, and both subjects recovered without intervention.

In the PF-dorzolamide/timolol group (*n* = 34), seven AEs in six subjects (17.6%) were reported. These included five AEs: eye pain (*n* = 2), eyelid edema (*n* = 1), keratitis (*n* = 1), and ocular hyperemia (*n* = 1), and abdominal pain (*n* = 1) and rash (*n* = 1). Six AEs were possibly related, and one was unlikely to be related to the study medication. AEs were mild (*n* = 5) or moderate (*n* = 2) in intensity. In all cases, AEs were resolved successfully, with one requiring hospitalization. No AEs were recorded in the P-latanoprost group (*n* = 36).

In the safety population (*n* = 107), the incidence rate of OSD-related AEs differed significantly between PF-tafluprost (*n* = 1; 2.7%), PF-dorzolamide/timolol (*n* = 4; 11.8%), and P-latanoprost (0%) groups (*p* = 0.042).

## 3. Discussion

This study compared two formulations of PF eye drops—tafluprost and dorzolamide/timolol—with P-latanoprost eye drops, which contain the preservative BAK (0.2 mg/mL). In addition to the fact that BAK is a known cause of corneal toxicities [13,15,17,20,21], chronic, low-grade inflammation of the ocular surface associated with BAK use, which is a risk factor for an increased incidence of AEs, lower tolerability, therapy failure, increased scarring, and subsequent filtration surgery failure, can occur with the long-term use of IOP-lowering therapies [31]. Consequently, the development of PF eye drops which can avoid the toxic effects associated with preservative-containing medications, may signal the way forward for lifelong IOP-lowering therapy [31]. According to previous in vitro and in vivo confocal microscopy studies, preservatives commonly contained in PGAs can potentially adversely affect meibomian gland function leading to further exaggeration of the risk for ocular surface dysfunction and, in that context, Guo et al. recently reported that diquafosol can circumvent this effect on meibomian gland dysfunction in patients treated with preserved PGAs comparable to being treated with preservative-free PGAs [32].

As combining all OSDI questionnaire responses into a single total score may hide differences between the various aspects of the disease, domain scores were evaluated. Mean changes in all three OSDI domains were significantly higher following treatment with PF-tafluprost and PF-dorzolamide/timolol, but not with P-latanoprost. Moreover, the magnitude of changes in OSDI with PF-tafluprost and PF-dorzolamide/timolol are consistent with achievement of a clinically significant response [32]. By splitting the OSDI scores into subscale domains concerning visual symptoms, functionality, and ocular discomfort due to environmental factors, our results suggest a differential effect of preservative-free formulations of tafluprost and dorzolamide/timolol over the preserved formulation of latanoprost with regard to the multidimensional aspect of patient-reported QoL.

In general, over the 12-week treatment period, our data showed a significant improvement in severity assessed by both overall and subscales scores of OSDI in PF-tafluprost and PF-dorzolamide/timolol treated patients, whilst P-latanoprost showed either no change or a worsening of scores. In comparison with the P-latanoprost arm, in the 12-week period, PF-tafluprost treatment allowed a significantly greater degree of improvement over P-latanoprost in overall vision-related QoL, primarily driven by an overlapping improvement in visual related functions (−13.05 points) and environmental triggers (−16.72 points). Karakus et al. [33] reported that total and all three subscale OSDI scores were significantly correlated with tear film parameters, i.e., corneal and conjunctival staining scores. In particular, the subscale for environmental trigger symptoms showed the strongest correlation with temporal conjunctival staining score at baseline [33] and, in the Japanese version of the OSDI, environmental trigger subscales showed significant negative correlation with maximum blink interval (MBI) [34]. This negative correlation could be explained by the fact that MBI has previously been reported to be significantly associated with tBUT and CFS. These results suggest that PF-tafluprost and PF-dorzolamide/timolol could have an advantage in reducing the risk of tear film dysfunction compared with P-latanoprost. Additionally, PF-tafluprost treatment can provide significant improvement over P-latanoprost in terms of disability for glaucoma patients performing daily activities that can be impacted by visual problems (e.g., reading, driving). Interestingly, a recent study revealed that visual-related function OSDI subscale scores were significantly associated with the number of drops per day of medication administered in glaucoma patients [30]. The observed improvement in visual-related QoL in the PF-tafluprost and PF-dorzolamide/timolol arm may be explained by the previous report from Rossi et al. which showed a potential reversibility of ocular surface toxicity caused by BAK when treatment was switched from preserved to preservative-free via examining the corneal nerves using in vivo confocal microscopy in glaucoma patients receiving IOP-lowering medications for 36 months [35].Use of the OSDI to assess QoL in patients with glaucoma is contentious. In a cross-sectional study of glaucoma patients, Mathews et al. concluded that the OSDI is a poor metric for measuring OSD in glaucoma as symptoms appear to be related largely to visual field loss [36], and Cveknel et al. reported that the OSDI failed to discriminate between treated and untreated glaucoma patients [37]. However, two studies reported that the OSDI was significantly increased in patients with glaucoma compared with controls [30,38]. In contrast to the study of Mathews et al. [36], the current study suggests that the OSDI might be a valid method to evaluate QoL in patients with glaucoma, with particular emphasis on ocular surface alterations.

Appraisal of the OSDI shows that it is reliable and internally consistent, and although it has a weak correlation with clinical dry eye tests, it is strongly correlated with other dry eye questionnaires and moderately correlated with artificial tear usage. In addition, the OSDI is an accurate discriminant between normal, mild/moderate, and severe dry eye disease [39,40]. In the present study, PF-tafluprost reduced the proportion of patients with severe dry eye disease by 29.8%, PF-dorzolamide/timolol by 20.6%, and P-latanoprost by 5.5%. The main QoL measure in the OSDI was related to the vision-related function domain [39], which was significantly improved by PF-tafluprost and PF-dorzolamide/timolol in the present study.

PF-tafluprost produced a significantly higher rate of PRO improvement (scored as improved/highly improved) compared with P-latanoprost (51.3% vs. 16.7%). A significant improvement in subjective satisfaction has also been reported in glaucoma patients switching from tafluprost containing BAK to PF-tafluprost [41]. Although the rate of PRO improvement with PF-dorzolamide/timolol (35.3%) was over two-fold higher than that with P-latanoprost (16.7%), statistical significance was not reached. A double crossover study found that the IOP-lowering effect of fixed-combination dorzolamide/timolol (Cosopt^®^) was non-inferior to that of P-latanoprost in patients with glaucoma [42]. Similarly, in glaucoma patients switched to open-label PF-tafluprost, IOP was maintained at the same level as P-latanoprost [43].

Higher OSD-related AEs were reported for PF-dorzolamide/timolol (*n* = 4; 11.8%), than PF-tafluprost (*n* = 1; 2.7%) and P-latanoprost (0%), but most AEs were mild and were resolved successfully. AEs reported for PF-dorzolamide/timolol are consistent with those found with dorzolamide/timolol containing preservative (Cosopt^®^), dorzolamide hydrochloride and/or timolol maleate [44]. The most common ocular treatment-related AEs in a study comparing dorzolamide/timolol (Cosopt^®^) with P-latanoprost were eye irritation (45.5% vs. 27.3%, respectively) and ocular hyperemia (4.5% vs.18.1%, respectively). Most AEs were mild in intensity and did not lead to study discontinuation [42], in line with the results reported in the current study. The two reported AEs (eye pruritus and blurred vision) for PF-tafluprost have been reported commonly (≥1/100 to <1/10) during clinical trials for this medication [43,45].

The strengths of the current study include the fact that it was conducted prospectively and that it was a multicenter study. However, there are some limitations, which include the nonrandomized nature of the study, as well as the relatively short study duration. Therefore, a randomized, controlled trial with a longer-term follow-up period would be warranted to further evaluate the comparative effects of the three treatments over an extended duration. In a previous single-center, prospective study, switching from ≥3 months of P-latanoprost to low preservative tafluprost for a minimum of 3 months was associated with significant improvements in fluorescein staining scores, tBUT, and subjective symptoms in patients with OSD [46]. In that particular study, patients were required to have a National Eye Institute (NEI) score above 1 [46], whereas the current study included patients with a corneal staining score of 3 or higher, and these differences in inclusion criteria may have contributed to differences in observed outcomes. The sample size was small for each treatment group. Further investigations with larger sample sizes are necessary to elucidate the potential effect of PF-tafluprost and PF-dorzolamide/timolol on OSD compared with BAK-containing latanoprost in glaucoma patients. Additionally, it is uncertain whether the study data are applicable to non-Korean OAG subjects (e.g., those of African or European descent with higher IOP) as our study participants were only Korean. Additionally, although not assessed in the current study, the importance of the complete assessment of IOP efficacy over a 24 h period has been highlighted previously for comparisons between PF-tafluprost and BAK-containing P-latanoprost in patients with open-angle glaucoma or ocular hypertension [47].

Additionally, it is possible that differences in the active constituents among three study treatments may have contributed to differential effects on the ocular surface scores noted at 12 weeks of follow-up. In a previous report by Moussa et al. [48], OSDI scores did not differ among various prostaglandin analogues, including latanoprost, and tafluprost. However, with respect to the PF-dorzolamide/timolol, the use of β-blockers in general was shown to increase corneal epithelial punctate erosion and associated with a shorter tBUT. Kuppens et al. reported that timolol caused a significant decrease in tBUT, regardless of the presence of preservative with respect to the control arm, which implied the possibility of timolol having a direct effect on the tear film [49]. Additionally, the impression cytology study by Kurna et al. [50] suggested that preserved and non-preserved timolol caused far more damage on the ocular surface than PGAs. Thus, timolol contained in the PF-dorzolamide/timolol used in the study might have impacted the observed ocular surface changes to a certain extent. Moreover, as the OSDI is a subjective instrument objectively assessing the ocular surface, its use to evaluate efficacy may have strengthened the study. However, the correlation between the OSDI and signs and symptoms of dry eye disease may be low and inconsistent in some patients [51].

Nevertheless, the significance of the current study is that symptoms that adversely affect visual-related QoL were improved from the patient’s point of view. Lastly, the current study did not include a washout period, as the primary objective of this study was to evaluate the differential OSD effect of each treatment intervention in glaucoma patients rather than assessing the IOP-lowering effects between the treatments, which have already been reported by a number of previous studies.

## 4. Materials and Methods

This was a multicenter, prospective, interventional, non-randomized controlled trial conducted on glaucoma patients with OSD who were previously treated with P-latanoprost. Patients were recruited from three hospitals in South Korea: Asan Medical Center, Chonnam National University Hospital, and Pusan National University Hospital. The trial was conducted from 18 May 2017 to 4 June 2018 and comprised observations at baseline, 4 weeks, and 12 weeks. Subjects who met the inclusion and exclusion criteria were enrolled into the following treatment groups based on the prescribed treatment according to usual clinical practice: PF-tafluprost, PF-dorzolamide/timolol, P-latanoprost. Subjects who were assigned to PF-tafluprost or PF-dorzolamide/timolol treatment group were switched from the use of P-latanoprost at baseline. Subjects who were enrolled into the P-latanoprost treatment group continued their treatment with P-latanoprost.

### 4.1. Inclusion and Exclusion Criteria

Eligible subjects were aged ≥19 to <80 years with glaucoma and OSD previously treated with P-latanoprost (containing the preservative, BAK), had an OSDI score of ≥23 and tear break-up time (tBUT) < 6 s or an OSDI score of ≤23, and the sum of the corneal scores following corneal fluorescein staining (CFS) was ≥3. Both eyes were enrolled when they met the inclusion criteria for the study. Exclusion criteria were: pregnant or lactating women or women of childbearing age who had a pregnancy plan; subjects with abnormal corneal pathologies that affect IOP measurement; corneal changes due to keratorefractive surgery; those who had undergone intraocular or laser surgery within 3 months of screening; use of artificial tears within 2 weeks before baseline; or use of cyclosporine and corticosteroids within 4 weeks before baseline.

### 4.2. Treatment

Patients were treated daily for 12 weeks with PF-tafluprost, PF-dorzolamide/timolol, or P-latanoprost containing the preservative (BAK) eye drops.

One drop of PF-tafluprost 15 μg/mL eye drops (Taflotan-S^®^) was administered once a day, with evening administration recommended. Preservative-free 20 mg/mL dorzolamide + 5 mg/mL timolol eye drops (PF-dorzolamide/timolol; Cosopt-S^®^) was applied to the conjunctival sac, one drop, twice daily to the affected eye(s). One drop of P-latanoprost (Xalatan^®^) 0.005% eye drops was administered once a day, with evening administration recommended.

The use of artificial tears or other glaucoma treatments was not permitted during the trial.

### 4.3. Efficacy Outcomes

The primary efficacy outcome was the change in OSDI score from baseline to 12 weeks. Secondary efficacy outcomes included changes in IOP from baseline to 12 weeks, and patient-reported satisfaction (PRO) at 12 weeks.

### 4.4. Efficacy Assessment

The OSDI is a valid and reliable instrument for assessing dry eye symptoms and their effects on vision-related function. It consists of a 12-item questionnaire containing three subscales for assessment of ocular symptoms, vision-related function, and environmental triggers. The OSDI has good to excellent test–retest reliability and effectively discriminates between normal, mild-to-moderate, and severe OSD [39,40].

Each item on the OSDI questionnaire is scored using a Likert scale: 0 (not at all), 1 (sometimes), 2 (about half the time), 3 (mostly), and 4 (always). The OSDI total score was calculated at each follow-up using the following equation: [(sum of scores for all questions answered) × 100]/[(total number of questions answered) × 4]. Total OSDI scores (ranging from 0 to 100) were categorized for disease severity as follows: normal = 0–12, mild = 13–22, moderate = 23–32, and severe = 33–100 [52]. In addition to the total OSDI score, the same formula was used to compute scores for the three sub-domains [39]. The three sub-domains were (1) ocular symptoms (specifically sensitivity to light, grittiness, sore/painful eyes, blurred vision, and poor vision) (questions 1–5); (2) visual problems impacting daily activities (reading, television viewing, computer work, and night-time driving) (questions 6–9); and (3) ocular discomfort triggered by environmental factors (wind, low humidity, and air conditioning) (questions 10–12).

Patient satisfaction was self-assessed (PRO) with patients answering the question, ‘How do you evaluate symptoms related to ocular surface disease (e.g., stiffness, dryness, foreign body sensation) compared with 12 weeks ago?’, scored as: highly improved, improved, similar, worse, or much worse. IOP was measured with a Goldman applanation tonometer at baseline, and at weeks 4 and 12.

### 4.5. Safety Assessment

All adverse events (AEs) following administration of the study drug were recorded and categorized by System Organ Class (SOC) and Preferred Term (PT), using the Medical Dictionary for Regulatory Activities (MedDRA).

### 4.6. Statistical Analysis

Continuous data were summarized as means (±standard deviation, SD) and categorical data as frequencies. These were analyzed using paired t-tests and chi-squared or Fisher’s exact tests, respectively. A *p*-value < 0.05 was considered to indicate statistical significance.

Power calculations showed that for 80% power and a significance level of 0.05 (two-sided test), a total of 102 subjects (34 per group) would be required. Accounting for a dropout rate of 15%, it was estimated that a total of 120 subjects (40 per group) would be needed for the study.

Efficacy was assessed using the Full Analysis Set (FAS), defined as patients who received the study drug and had at least one evaluable efficacy endpoint. To account for missing value correction, the FAS was further divided into FAS1 (using the last observation carried forward [LOCF] method) and FAS2 (missing values not replaced).

The per-protocol set (PPS) was defined as subjects in the FAS who completed the trial according to the study protocol, excluding subjects who dropped out of the clinical trial without completing the period specified in the protocol, those who received a combination-prohibited drug, study inclusion/exclusion criteria violators, or any other cases considered a violation of the protocol which were considered to possibly affect the effectiveness evaluation. Safety was assessed using the safety set, defined as all enrolled subjects who received clinical trial drugs.

Statistical analyses were performed using the program SAS (Statistical Analysis Software 9.4, SAS Institute Inc, Cary, NC, USA) and visualizations were performed with R version 4.0.3 (R Foundation for Statistical Computing, Vienna, Austria).

## 5. Conclusions

PF-tafluprost and PF-dorzolamide/timolol demonstrated statistically and clinically significant improvements in patient-reported, visual-related QoL based on the OSDI questionnaire, compared with preservative-containing latanoprost, whilst having a comparable IOP reduction effect on Korean glaucoma patients with OSD.

## Figures and Tables

**Table 1:** Patient demographics at baseline in the FAS1 (*n* = 107).

Characteristic	PF-Tafluprost (*n* = 37)	PF-Dorzolamide/Timolol (*n* = 34)	P-Latanoprost (*n* = 36)
Age, years: mean (SD)	63.2 (13.2)	62.1 (14.0)	63.0 (13.1)
Male: n (%)	15 (40.5)	17 (50)	14 (38.9)
Intraocular pressure, mmHg: mean (SD)	13.8 (2.3)	13.1 (3.4)	13.1 (2.7)
Prior medication *: n	67	63	96
Anti-anginal	7 (10.5)	1 (1.6)	0
Anti-asthmatic/COPD	0	0	10 (10.4)
Dyslipidemic	7 (10.5)	7 (11.1)	8 (8.3)
Oral anti-diabetic	0	8 (12.7)	10 (10.4)

* Patients may have received more than one prior medication. FAS1, full analysis set 1; P, preservative-containing; PF, preservative-free; SD, standard deviation.

**Table 2:** Change in OSDI from baseline to 12-week follow-up in all patients in the FAS1 population (*n* = 107).

OSDI Questionnaire	PF-Tafluprost		PF-Dorzolamide/Timolol		P-Latanoprost	
	Mean (SD)	*p*-Value †	Mean (SD)	*p*-Value †	Mean (SD)	*p*-Value †
Total score	–9.5 (18.9)	0.0042 *	–10.5 (19.8)	0.0038 *	–1.5 (18.3)	0.6256
Dry eye symptoms	–6.8 (19.4)	0.0412 *	–10.2 (20.3)	0.0063 *	–3.3 (14.7)	0.1834
Visual-related function	–12.2 (23.5)	0.0047 *	–11.1 (26.5)	0.0291 *	–0.9 (28.8)	0.8610
Environmental triggers	–13.9 (29.6)	0.0088 *	–14.0 (22.9)	0.0013 *	1.2 (32.9)	0.8318

† Calculated using paired t-tests for change in OSDI from baseline to follow-up for each treatment group. * *p* < 0.05. FAS1, full analysis set 1; OSDI, ocular surface disease index; PF, preservative-free; SD, standard deviation.

**Table 3:** Comparison of PF-tafluprost and PF-dorzolamide/timolol with P-latanoprost with respect to the between-group differences in the change in OSDI from baseline to 12-week follow-up in the FAS1 population (*n* = 107).

OSDI Questionnaire	PF-Tafluprost–P-Latanoprost (a)	PF-Dorzolamide/Timolol–P-Latanoprost (b)	*p*-Value †	
	Mean BGD (SD)	Mean BGD (SD)	(a)	(b)
Total score	8.0 (18.6)	9.0 (19.0)	0.0707	0.0510
Dry eye symptom domain	3.4 (17.3)	6.8 (17.7)	0.3998	0.1110
Visual-related function domain	11.3 (26.2)	10.2 (27.7)	0.0821	0.1487
Environmental triggers domain	15.1 (31.3)	15.2 (28.5)	0.0474 *	0.0299 *

† Two-sample t-test. * *p* < 0.05 vs. P-latanoprost. BGD, between-group-difference in change in score from baseline to 12 weeks; FAS1, full analysis set 1; OSDI, ocular surface disease index; P, preservative-containing; PF, preservative-free; SD, standard deviation.

**Table 4:** Patient-reported treatment satisfaction in the FAS2 and PPS populations †.

Population	Treatment Group	Highly Improved	Improved	Similar	Worse	Much Worse
		*n* (%)	*n* (%)	*n* (%)	*n* (%)	*n* (%)
FAS2 (*n* = 105) ††	PF-tafluprost (*n* = 35)	6 (16.2)	13 (35.1)	15 (40.5)	1 (2.7)	0
	PF-dorzolamide/timolol (*n* = 34)	2 (5.9)	10 (29.4)	20 (58.8)	0	2 (5.9)
	P-latanoprost (*n* = 36)	1 (2.8)	5 (13.9)	24 (66.7)	5 (13.9)	1 (2.8)
PPS (*n* = 97) †††	PF-tafluprost (*n* = 32)	6 (18.8)	11 (34.4)	14 (43.8)	1 (3.1)	0
	PF-dorzolamide/timolol (*n* = 30)	2 (6.7)	10 (33.3)	17 (56.7)	0	1 (3.3)
	P-latanoprost (*n* = 35)	1 (2.9)	5 (14.3)	24 (68.6)	4 (11.4)	1 (2.9)

† Only FAS2 and PPS population results are presented. FAS1 population results could not be analyzed due to the inability of using the last-observation-carried-forward method, as PROs were only measured once at the end of follow-up (visit 3) and data were missing from two patients who were excluded during the trial. †† For patients with highly improved/improved PROs: PF-tafluprost vs. P-latanoprost, *p* = 0.007; PF-dorzolamide/timolol vs. P-latanoprost, *p* = 0.078. ††† For patients with highly improved/improved PROs: PF-tafluprost vs. P-latanoprost, *p* = 0.016; PF-dorzolamide/timolol vs. P-latanoprost, *p* = 0.102. FAS, full analysis set; P, preservative-containing; PF, preservative-free; PPS per protocol set; PRO, patient-reported outcomes.

**Table 5:** Change in intraocular pressure from baseline to 12 weeks in the FAS1 population.

IOP	Mean (SD) IOP (mmHg)		
	PF-Tafluprost (*n* = 37)	PF-Dorzolamide/Timolol (*n* = 34)	P-Latanoprost (*n* = 36)
Baseline	13.76 (2.31)	13.09 (3.41)	13.11 (2.70)
12-weeks	13.73 (3.47)	13.44 (2.72)	13.08 (2.26)
Change from baseline	−0.03 (2.76)	0.35 (3.66)	−0.03 (1.87)

PF-tafluprost vs. P-latanoprost: *p* = 0.999; PF-dorzolamide/timolol vs. P-latanoprost: *p* = 0.590. FAS1, full analysis set 1; IOP, intraocular pressure; P, preservative-containing; PF, preservative-free; SD, standard deviation.

## Data Availability

Data is contained within the article and supplementary files.

## Supplementary Materials

The following are available online at https://www.mdpi.com/article/10.3390/ph15020201/s1, Figure S1: Ocular Surface Disease Index (OSDI) severity categories in each treatment arm at baseline and 12-week follow-up in the per protocol (PP) population (*n* = 97), Table S1: Change in OSDI from baseline to 12-week follow-up in the PPS population (*n* = 97), Table S2: OSDI total and subgroup scores at baseline and 12-week follow-up in the FAS1 population (*n* = 107), Table S3: OSDI total and subdomain scores at baseline and 12-week follow-up in the FAS2 population (*n* = 105), Table S4: OSDI total and subgroup scores at baseline and 12-week follow-up in the PPS population (*n* = 97), Table S5: Comparison of PF-tafluprost and PF-dorzolamide/timolol with P-latanoprost with respect to the between-group differences in the change in OSDI from baseline to 12-week follow-up in the PPS populations.
