# Supplementary material for: Efficacy, Safety and Patient-Reported Outcomes with Preservative-Free (PF) Tafluprost or PF-Dorzolamide/Timolol Compared with Preserved Latanoprost: A Prospective Multicenter Study in Korean Glaucoma Patients with Ocular Surface Disease
Source: Pharmaceuticals (Basel). 2022 Feb 7;15(2):201. doi: 10.3390/ph15020201 (PMC8874539; doi:10.3390/ph15020201)

Supplementary Tables and Figures

Supplementary Table S1. Change in OSDI from baseline to 12-week follow-up in the PPS population (N = 97)

OSDI questionnaire	PF-tafluprost		PF-dorzolamide/timolol		P-latanoprost	
	Mean (SD)	P-value†	Mean (SD)	P-value†	Mean (SD)	P-value†
Total score	–11.963 (17.797)	0.0006*	–11.915 (20.249)	0.0031*	–0.914 (18.235)	0.7686
Dry eye symptoms	–8.125 (19.665)	0.0261*	–11.500 (20.559)	0.0047*	–2.714 (14.468)	0.2748
Visual-related function	–16.236 (21.895)	0.0004*	–13.061 (27.345)	0.0223*	–0.912 (29.224)	0.8611
Environmental triggers	–17.361 (27.662)	0.0018*	–15.374 (23.229)	0.0013*	2.696 (32.159)	0.6282

†Calculated using paired t-tests for change in OSDI from baseline to follow-up for each treatment group.
* P<0.05
OSDI, Ocular Surface Disease Index; *P*, preservative-containing; *PF*, preservative-free; *PPS*, per-protocol set; *SD*, standard deviation

Supplementary Table S2. OSDI total and subgroup scores at baseline and 12-week follow-up in the FAS1 population (N = 107)

Visit	OSDI questionnaire	All subjects (N = 107)	PF-tafluprost (a) (N = 37)	PF-dorzolamide/timolol (b) (N = 34)	P-latanoprost (c) (N = 36)	P-value†	
		Mean (SD)	Mean (SD)	Mean (SD)	Mean (SD)	a *vs.* c	b *vs.* c
Baseline	Total score	32.481 (23.252)	35.392 (23.004)	29.821 (23.867)	32.003 (23.238)	0.5332	0.6996
	Dry eye symptoms	30.234 (23.506)	30.541 (22.323)	31.029 (27.737)	29.167 (20.822)	0.7866	0.7508
	Visual-related function	34.917 (29.453)	38.393 (28.825)	28.831 (26.826)	36.887 (32.257)	0.8385	0.2804
	Environmental triggers	36.378 (31.046)	42.940 (33.848)	31.439 (25.454)	34.286 (32.513)	0.2759	0.6902
Follow-up	Total score	25.343 (22.273)	25.895 (20.631)	19.281 (21.254)	30.500 (23.985)	0.3817	0.0426*
	Dry eye symptoms	23.551 (20.547)	23.784 (19.558)	20.882 (22.545)	25.833 (19.839)	0.6581	0.3322
	Visual-related function	26.362 (27.404)	25.810 (24.363)	17.551 (23.438)	35.238 (31.467)	0.1619	0.0110*
	Environmental triggers	27.594 (30.104)	29.398 (31.068)	18.382 (24.685)	34.491 (32.315)	0.4977	0.0226*

†Calculated using paired t-tests for comparisons with the P-latanoprost group
* P<0.05
FAS1, full analysis set 1, *OSDI*, Ocular Surface Disease Index; *P*, preservative-containing; *PF*, preservative-free; *SD*, standard deviation

Supplementary Table S3. OSDI total and subdomain scores at baseline and 12-week follow-up in the FAS2 population (N = 105)

Visit	OSDI questionnaire	All subjects (N = 105)	PF-tafluprost (a) (N = 35)	PF-dorzolamide/timolol (b) (N = 34)	P-latanoprost (c) (N = 36)	P-value†	
		Mean (SD)	Mean (SD)	Mean (SD)	Mean (SD)	a *vs.* c	b *vs.* c
Baseline	Total score	32.862 (23.243)	36.700 (22.772)	29.821 (23.867)	32.003 (23.238)	0.3928	0.6996
	Dry eye symptoms	30.667 (23.494)	31.857 (22.165)	31.029 (27.737)	29.167 (20.822)	0.5997	0.7508
	Visual-related function	35.374 (29.522)	39.962 (28.785)	28.831 (26.826)	36.887 (32.257)	0.6822	0.2804
	Environmental triggers	36.683 (31.137)	44.240 (34.021)	31.439 (25.454)	34.286 (32.513)	0.2183	0.6902
Follow-up	Total score	25.289 (22.445)	25.766 (21.100)	19.281 (21.254)	30.500 (23.985)	0.3808	0.0426*
	Dry eye symptoms	23.524 (20.743)	23.714 (20.123)	20.882 (22.545)	25.833 (19.839)	0.6564	0.3322
	Visual-related function	26.328 (27.641)	25.674 (24.977)	17.551 (23.438)	35.238 (31.467)	0.1674	0.0110*
	Environmental triggers	27.484 (30.183)	29.167 (31.382)	18.382 (24.685)	34.491 (32.315)	0.4872	0.0226*

†Paired t-test
* P<0.05
FAS2, full analysis set 2, *OSDI*, Ocular Surface Disease Index; *P*, preservative-containing*; PF*, preservative-free; *SD*, standard deviation

Supplementary Table S4. OSDI total and subgroup scores at baseline and 12-week follow-up in the PPS population (N = 97)

Visit	OSDI questionnaire	All subjects (N = 97)	PF-tafluprost (a) (N = 32)	PF-dorzolamide/timolol (b) (N = 30)	P-latanoprost (c) (N = 35)	P-value†	
		Mean (SD)	Mean (SD)	Mean (SD)	Mean (SD)	a *vs.* c	b *vs.* c
Baseline	Total score	32.501 (22.589)	37.472 (22.153)	29.743 (24.392)	30.320 (21.236)	0.1821	0.9192
	Dry eye symptoms	29.794 (22.695)	31.563 (21.419)	30.333 (27.821)	27.714 (19.187)	0.4407	0.6657
	Visual-related function	35.787 (29.248)	42.083 (28.494)	29.784 (27.854)	34.975 (30.737)	0.3463	0.5001
	Environmental triggers	36.613 (31.036)	45.565 (33.900)	32.040 (26.659)	32.353 (30.893)	0.1052	0.9661
12 weeks	Total score	24.540 (21.362)	25.509 (18.771)	17.828 (20.384)	29.406 (23.406)	0.4577	0.0390*
	Dry eye symptoms	22.577 (19.232)	23.438 (18.159)	18.833 (20.115)	25.000 (19.478)	0.7359	0.2147
	Visual-related function	25.621 (26.913)	25.538 (23.887)	16.667 (24.308)	33.333 (29.822)	0.2521	0.0193*
	Environmental triggers	27.257 (29.795)	28.763 (29.312)	17.778 (24.830)	34.048 (32.676)	0.4940	0.0293*

†P values were calculated using paired t-tests for comparisons with the P-latanoprost group.
* P<0.05
OSDI, , Ocular Surface Disease Index; *P*, preservative-containing; *PF*, preservative-free; *PPS*, per-protocol set; *SD*, standard deviation

Supplementary Table S5. Comparison of PF-tafluprost and PF-dorzolamide/timolol with P-latanoprost with respect to the between-group differences in the change in OSDI from baseline to 12-week follow-up in the PPS populations

**Population**	**OSDI questionnaire**	**PF-tafluprost – P-latanoprost (a)**	**PF-dorzolamide/timolol – P-latanoprost (b)**	**P-value†**	
		**Mean BGD (SD)**	**Mean BGD (SD)**	**(a)**	**(b)**
PPS	Overall	11.048 (18.027)	11.001 (19.188)	0.0147*	0.0245*
	Dry eye symptom domain	5.411 (17.144)	8.786 (17.537)	0.2015	0.0483*
	Visual-related function domain	15.324 (26.005)	12.149 (28.401)	0.0251*	0.1108
	Environmental triggers domain	20.057 (30.139)	18.070 (28.410)	0.0100*	0.0145*

†Two sample T-test
* P<0.05 compared with P-latanoprost
BGD between-group difference in change in score from baseline to 12 weeks; *OSDI*, Ocular Surface Disease Index; *P*, preservative-containing; *PF*, preservative-free; *PPS*, per-protocol set; *SD*, standard deviation

Supplementary Figure S1. Ocular Surface Disease Index (OSDI) severity categories in each treatment arm at baseline and 12-week follow-up in the per protocol (PP) population (N = 97). Taflotan-S = preservative-free tafluprost; Cosopt-S = preservative-free dorzolamide + timolol; Latanoprost = preservative-containing latanoprost

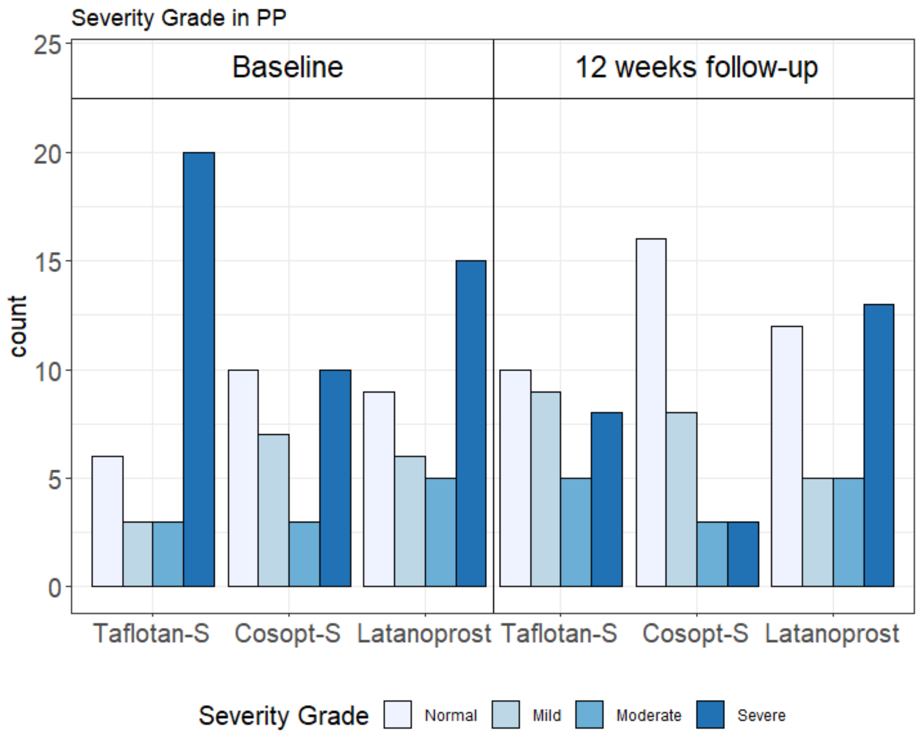

Supplement: Supplementary file 1 [file pharmaceuticals-15-00201-s001.zip › pharmaceuticals-1539739-supplementary.pdf]
